# Aldosterone and Cardiovascular Risk Across the Lifespan

**DOI:** 10.3390/metabo15080553

**Published:** 2025-08-17

**Authors:** Roshan A. Ananda, Trevor A. Mori, Jun Yang

**Affiliations:** 1 Department of General Medicine, Box Hill Hospital, Melbourne, VIC 3128, Australia; roshan.ananda21@alumni.imperial.ac.uk; 2 Medical School, Royal Perth Hospital Unit, University of Western Australia, Perth, WA 6000, Australia; trevor.mori@uwa.edu.au; 3 Endocrine Hypertension Group, Centre for Endocrinology and Reproductive Health, Hudson Institute of Medical Research, Melbourne, VIC 3168, Australia; 4 Department of Molecular and Translational Science, Monash University, Melbourne, VIC 3168, Australia; 5 Department of Medicine, Monash University, Melbourne, VIC 3168, Australia

**Keywords:** aldosterone, primary aldosteronism, cardiovascular risk

## Abstract

Aldosterone excess, particularly in the context of primary aldosteronism, is associated with adverse cardiovascular outcomes. Historically considered a condition of resistant hypertension with hypokalaemia, patients with primary aldosteronism often experienced prolonged diagnostic delay with significant end-organ damage involving the renal, cardiovascular, and central nervous systems at diagnosis. Emerging research has revealed a wide spectrum of renin-independent aldosteronism, ranging from subclinical disease with normal or mildly elevated BP to overt disease marked by resistant hypertension and cardiovascular complications. Subclinical forms of primary aldosteronism have been identified across all age groups, and it is increasingly linked to early signs of adverse cardiac remodelling, even in young adults. Notably, adverse cardiac remodelling was independent of blood pressure. Furthermore, primary aldosteronism confers excess cardiovascular morbidity and mortality compared to blood-pressure-matched essential hypertension. Importantly, these risks can be mitigated through timely diagnosis and treatment with mineralocorticoid receptor antagonists. In this narrative review, we explore the cardiovascular consequences of aldosterone excess, discuss the pathophysiological mechanisms underlying cardiac remodelling, and examine the implications of renin-independent aldosteronism for cardiovascular risk across the lifespan.

## 1. Introduction

Aldosterone is a steroid hormone secreted by the adrenal glands [1]. It activates the mineralocorticoid receptors (MR) to regulate blood pressure (BP) and maintain electrolyte homeostasis [2]. While MRs are best known for their role in the renal tubules, they are also widely expressed across various tissues, including the cardiovascular system, adipose tissue and immune cells, where inappropriate activation may lead to tissue inflammation and fibrosis [3]. Thus, in the pathological state of autonomous, renin-independent aldosterone production, a condition known as primary aldosteronism (PA), affected individuals face an increased risk of renal, metabolic, and cardiovascular diseases [4].

Traditionally considered a disease of resistant hypertension with hypokalaemia, emerging research has revealed a wide spectrum of renin-independent aldosteronism, ranging from subclinical disease with normal or mildly elevated BP to more severe presentations involving resistant hypertension and cardiovascular complications [4,5,6,7,8]. Although florid disease is commonly associated with cardiovascular morbidity and mortality, the heightened cardiovascular risk begins with subclinical PA which may begin in young adulthood [4,9]. Young adults with subclinical PA are at risk of developing adverse structural and functional changes in their left ventricles, including left ventricular mass index and impaired mid-wall fractional fibre shortening in the left ventricle [9,10]. These excess risks are independent of systolic BP, and can lead to more severe and excessive morbidity for the degree of hypertension in the middle-aged and elderly population, in the form of heart failure, arrhythmias, myocardial infarction and strokes [4,11].

In addition to heightened cardiovascular risk, aldosterone excess has been implicated in increased risk of renal and metabolic complications, as well as reduced quality of life [12]. This review will focus on the cardiovascular aspects of aldosterone excess and explore the relationship between aldosterone excess and cardiovascular risk across the lifespan.

## 2. Aldosterone: A Hormone in the Cardiovascular System

**Figure 1:**
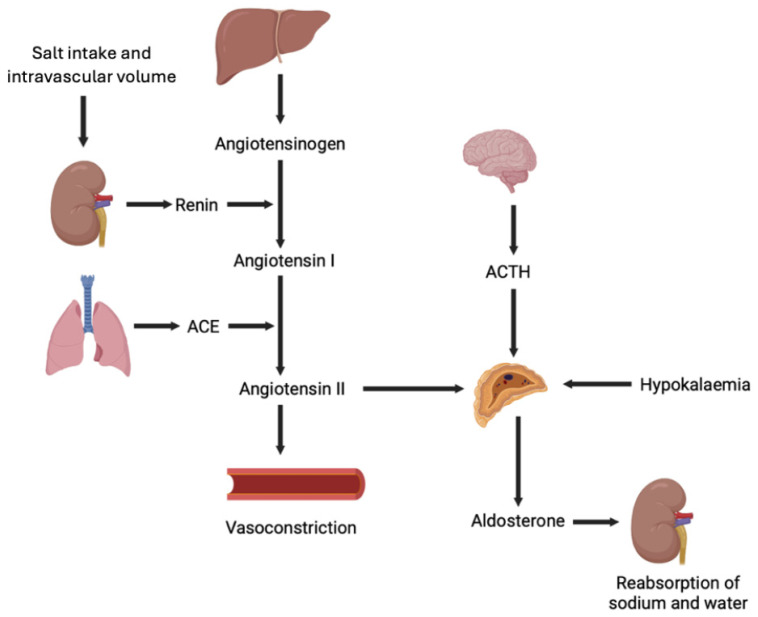
Schematic of the regulation of aldosterone production by renin–angiotensin–aldosterone system, circulating potassium, and ACTH. ACE, angiotensin-converting enzyme; ACTH, adrenocorticotropic hormone.

Aldosterone is synthesised in the zona glomerulosa of the adrenal cortex [1]. Its production is tightly regulated by angiotensin II, circulating potassium, and adrenocorticotropic hormone (ACTH) (Figure 1) [13].

Aldosterone activates the MR, a nuclear transcription factor that regulates gene expression, to mediate downstream physiological, or pathophysiological effects [14]. MRs are widely expressed in a range of tissues, including renal tubules epithelium, salivary epithelium, colonic epithelium, vascular smooth muscle cells, cardiomyocytes, cardiac fibroblasts and the central nervous system [3,15]. In the renal tubules, which is considered the primary site of MR action, aldosterone promotes sodium retention and water reabsorption, maintaining intravascular volume and regulating BP [14]. During this process, potassium is excreted by the kidneys and acid–base balance is maintained [14].

In the cardiovascular system, aldosterone modulates sodium and potassium balance in cardiomyocytes through the sodium-potassium pump (Na^+^-K^+^-ATPase) [16]. This process maintains the proper intracellular ion gradients which are critical for cardiac contraction and action potential propagation [16]. Additionally, aldosterone increases intracellular calcium levels in cardiomyocytes by activating the voltage-gated L-type and T-type calcium channels and stimulating calcium release from the sarcoplasmic reticulum [17]. Elevated intracellular calcium exerts positive inotropic effects, enhancing cardiac contractility [17].

Aldosterone also plays an important role in cardiac remodelling, often as a compensatory response to stress or injury, such as hypertension, heart failure or myocardial infarction [18]. Aldosterone stimulates cardiac fibroblasts to produce collagen and other extracellular matrix proteins for deposition at sites of damage, which in the long-term leads to cardiac fibrosis [18,19]. MR activation in cardiomyocytes can also promote cardiac hypertrophy by increasing protein synthesis and cell growth through the activation of mitogen-activated protein kinase (MAPK) pathways [20].

In vascular smooth muscle cells, aldosterone regulates vascular tone and BP by increasing intracellular calcium concentrations through the influx of calcium via the L-type calcium channel and mobilisation of calcium from internal calcium storage [21,22]. The intracellular and extracellular calcium movement are essential for smooth muscle contraction and also contribute to vasoconstriction, increasing vascular resistance and BP [21,23]. Additionally, aldosterone promotes vasoconstriction indirectly by stimulating the secretion of endothelin-1, a potent vasoconstrictor [23].

Aldosterone plays a central role in promoting vascular modelling in response to stress or injury [18,24]. It induces proliferation of vascular smooth muscle cells, leading to intimal thickening of the blood vessels [25]. Aldosterone can also stimulate the production and deposition of collagen and extracellular matrix proteins in vessel walls, resulting in vascular fibrosis and arterial stiffness [18,25].

Serum aldosterone concentration is maintained through a negative feedback loop of the renin–angiotensin–aldosterone system (RAAS) (Figure 1) [1]. The kidneys release renin in response to decreased extracellular fluid volume, low BP or the activation of the sympathetic nervous system [26]. Renin acts as an enzyme to convert angiotensinogen into angiotensin I, which is then converted to angiotensin II by the angiotensin-converting enzyme [27]. Apart from its role as a vasoconstrictor, angiotensin II stimulates the adrenal glands to secrete aldosterone to increase extracellular volume and restore normal BP [1,3,26]. The increase in volume then exerts negative feedback on renin production and the RAAS pathway, leading to maintenance of normal BP [1,3].

In the pathological condition of PA, there is autonomous aldosterone production from one or both adrenal glands, largely independent of the RAAS feedback loop [1,3]. Having aldosterone production that is disproportionate to the level of renin leads to an elevated aldosterone-to-renin ratio (ARR), which defines a positive screening test for PA. Long term exposure to aldosterone excess, which is inappropriate for sodium and volume status, promotes adverse tissue remodelling in the cardiovascular system and contributes to heightened cardiovascular risk across the lifespan [4].

## 3. Aldosterone and Cardiovascular Risk in Early Life

Disturbances in aldosterone signalling during early life may have long-term implications for cardiovascular health, as aldosterone excess is a known risk factor for cardiovascular diseases [4]. However, to our knowledge, no studies have specifically investigated whether infants with elevated serum aldosterone levels are at increased risk of developing cardiovascular diseases later in life. Studies have shown that birthweight is inversely associated with serum aldosterone levels and BP in children and adolescents, indicating a potential link between perinatal factors and long-term cardiovascular outcomes [28]. On the other end of the spectrum, conditions with low serum aldosterone levels in infancy, such as congenital adrenal hyperplasia, can result in severe complications, including life-threatening arrhythmias and heart failure due to salt-wasting and concurrent glucocorticoid deficiency [29]. Furthermore, individuals with congenital adrenal hyperplasia have a higher risk of cardiovascular diseases when they reach adulthood [30].

Although hypertension is uncommon among children, sporadic cases of PA can occur during childhood [31]. If left untreated, they will have more years accumulated for aldosterone-mediated adverse effects on their cardiovascular health [31]. PA is a potentially curable disease, and there is no difference in the diagnosis and treatment between children and adults [31,32,33]. Even in the absence of a formal diagnosis of PA, aldosterone levels per se have been positively correlated with adverse left ventricular (LV) remodelling in children [34]. Among children aged 7 to 18 years (n = 102) from the United States, there was a positive association between serum aldosterone concentration and left ventricular mass index (LVMI), but not ambulatory systolic BP [34]. This study suggests that aldosterone-mediated adverse cardiac remodelling can occur as early as childhood, and that this relationship is not related to BP changes [34]. Furthermore, aldosterone has been found to mediate the positive association between obesity and LVMI [35].

## 4. Aldosterone and Cardiovascular Health During Adolescence and Young Adulthood

The association between markers of aldosterone excess and adverse cardiovascular health has been demonstrated in adolescence and young adulthood [9,36]. In the Raine Study, a pregnancy-birth cohort from Western Australia, ARR at 17 years of age (n = 871) was positively associated with systolic BP in males at age 17 years and females at age 27 years [9]. In the same study at age 27 years (n = 758), serum aldosterone concentration was positively associated with LVMI among males, while ARR was positively associated with LVMI among females. These associations were independent of systolic BP [9]. Although the aldosterone profile was considered normal in most participants, and the changes in LVMI were subtle and not yet consistent with LV hypertrophy, these early adverse changes in LV remodelling likely predispose to future cardiovascular risk [9,37]. Based on data from the Framingham Heart Study which examined cardiovascular risk with changes in LVMI, the 27 year old participants from the Raine Study would experience a 3.0% increase in cardiovascular risk with every 100 pmol/L increase in aldosterone concentration (reference range ~ 200–800 pmol/L) in males; and a 3.2% increase in risk with every 10 pmol/mU increase in ARR (reference < 70) in females [37].

Aldosterone-mediated adverse LV remodelling has been observed in other young adult populations [10,38,39]. Among Black adolescents (aged 15–19 years), there was a positive association between serum aldosterone and LVMI, but this association was thought to be mediated by aldosterone-related sodium retention and volume-mediated increase in BP [39]. Among young adults (aged 20–40 years) with normal or mildly elevated BP, aldosterone was correlated with adverse structural (LV mass) and functional changes (impaired mid-wall fractional fibre shortening) in the left ventricles [10]. Lower renin, a marker of excess MR activation, was associated with LVMI in young Black women (aged 20–30 years) from low socio-economic backgrounds, but not in the overall cohort [38].

The association between ARR and BP has been demonstrated in several young adult populations [40,41]. In a European population-based study of young adults (aged 25–41 years), ARR was positively associated with conventional and 24 h ambulatory BP measurements, even at levels that are considered normal (ARR < 70) [41]. Conventional and 24 h ambulatory BP increased by 1.68 mmHg and 2.40 mmHg, respectively, with every 1-unit increase in log-transformed ARR [41]. A similar association was also shown in young Black women (aged 18–50 years) on controlled sodium diets [40].

Collectively, these studies provide evidence for the relationship between aldosterone and cardiovascular health from a young age.

## 5. Subclinical Primary Aldosteronism and Cardiovascular Risk Among Adults

Subclinical primary aldosteronism (PA), defined as a condition with elevated ARR or suppressed renin but which does not meet the full diagnostic criteria for PA, has been reported in older adults who are more likely to suffer from more severe cardiovascular complications compared to young adults [7,8,11,42,43]. In a population-based cohort study of 1284 middle-aged participants (40–69 years) from Canada, subclinical PA was associated with increased arterial stiffness and incident hypertension [11]. This phenotype was also associated with adverse cardiac remodelling, including increased indexed maximum left atrial volume, LVMI, and LV remodelling index [11]. Participants with subclinical PA had higher odds of developing LV hypertrophy, even among those with normal BP, highlighting the BP-independent adverse effect of aldosterone excess on the cardiovascular system [11].

The Jackson Heart Study, a cohort study of African Americans, has shown that aldosterone partially mediates the inverse association between ideal cardiovascular health and incidence of cardiovascular diseases [42]. In that study, ideal cardiovascular health was assessed using a dichotomous metrics (ideal vs. intermediate/poor) which included smoking status, diet intake, physical activity, body mass index, and total cholesterol level [42]. Similarly, data from the Framingham Offspring Study also demonstrated that aldosterone concentration was inversely associated with ideal cardiovascular health, and this relationship heightened the risk for subclinical as well as incident cardiovascular diseases [43]. In the Atherosclerosis Risk in Communities (ARIC) study of 4547 adults without prevalent heart failure, higher aldosterone concentration was associated with higher incidence of atrial fibrillation and heart failure [6]. The Multi-Ethnic Study of Atherosclerosis (MESA), reported a dose–response relationship between serum aldosterone concentration and the severity of coronary artery calcification, with the relationship more prominent among those with suppressed plasma renin activity [7]. Their findings support the possible causal relationship between aldosterone excess and subclinical atherosclerosis [7]. Among hypertensive patients with white matter lesions in their central nervous system, serum aldosterone concentration was positively associated with new-onset stroke and intracerebral haemorrhage [44].

## 6. Aldosterone Excess in Adulthood: A Multidimensional Disease Beyond Blood Pressure

Aldosterone excess is highly prevalent in the general population and increasingly recognised as the most common cause for secondary hypertension [45]. Based on a cohort study from France, 619 of 2090 young participants aged 18 to 40 years with confirmed hypertension (29.6%) had secondary hypertension, and 54.8% (n = 339) were diagnosed with PA [45].

PA is a state of autonomous renin-independent aldosterone excess that causes hypertension and increased potassium excretion. Traditionally, PA is diagnosed when plasma or urinary aldosterone concentration fail to be suppressed below a laboratory-specific threshold by either sodium loading (through intravenous saline, oral salt, or fludrocortisone) or captopril. Historically considered as an uncommon disease that causes resistant hypertension and hypokalaemia, individuals with PA often suffer from prolonged diagnostic delay [46] and in many cases only after the manifestation of end-organ damage such as renal failure or stroke [47].

**Figure 2:**
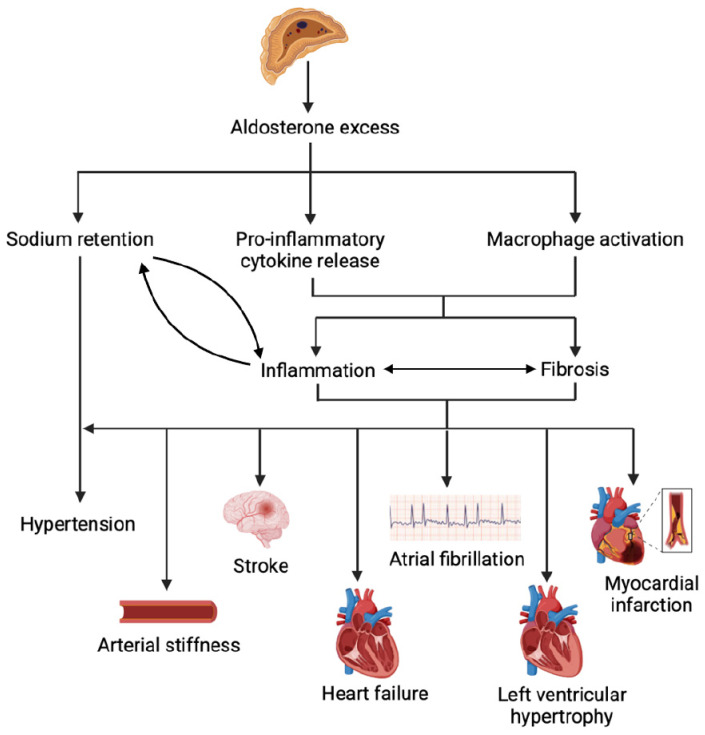
Mechanisms and complications of aldosterone excess.

PA confers a higher risk of cardiovascular complications (Figure 2) [4,48]. In a meta-analysis of 13,122 individuals from 31 studies, individuals with PA (n = 3838) have 3.5 times higher odds of developing atrial fibrillation, 2.6 times higher odds of new-onset stroke, 2.3 times higher odds of LV hypertrophy, and two-fold higher odds of incident coronary artery disease and heart failure, compared to those with essential hypertension (n = 9284), even when matched for BP [4]. These excess cardiovascular risks highlight the independent pathological effects of aldosterone excess, in addition to the mechanical stress from elevated systolic BP, on the heart and vasculature [4,48].

### 6.1. Hypertension and Arterial Stiffness

Hypertension as a consequence of PA is mostly caused by MR-mediated increase in sodium resorption and extracellular fluid volume expansion [1,49,50]. Other mechanisms that may contribute to aldosterone-induced hypertension are arterial stiffness and increased vasoconstrictive effects [23]. Increased oxidative stress due to aldosterone excess increases the susceptibility of blood vessel walls to injury [51]. As a compensatory mechanism, aldosterone will stimulate vascular smooth muscle cell proliferation and fibrosis with consequent stiffening of the arteries and increased BP [51,52]. Aldosterone excess also stimulates the production of endothelin-1, a potent vasoconstrictor, leading to increased vasoconstrictive effects [23]. Increased intracellular calcium concentration in vascular smooth muscle cells from aldosterone-mediated calcium influx through L-type calcium channels and mobilisation from internal calcium storage increases vascular resistance, and thereby increasing BP [21,23].

Aldosterone can also exacerbate arterial stiffness via endothelial dysfunction, which plays an important role in the progression of atherosclerosis [51,53,54]. In young adults (aged 18–50 years; n = 972), plasma aldosterone and ARR were inversely associated with flow-mediated dilation (FMD) of the brachial artery, which is a marker for endothelial dysfunction [53]. In a prospective study from Taiwan, participants with PA (n = 67) had more severe arterial stiffness, as measured by pulse wave velocity, than those with BP-matched essential hypertension [54]. In the same study, markers for vascular dysfunction improved after 6 months of targeted treatment for PA.

### 6.2. Myocardial Infarction and Stroke

An increased risk of stroke and myocardial infarction in individuals with PA may be attributed to the combined effects of endothelial dysfunction and atherosclerosis [4,48,51,55]. In addition to stimulating endothelin production, aldosterone excess reduces nitric oxide bioavailability by increasing the transcription of NAD(P)H oxidase in endothelial cells, promoting the production of reactive oxygen species that contribute to endothelial dysfunction [51,55]. Aldosterone also activates NAD(P)H oxidase in macrophages, facilitating the formation of foam cells and the development of atherosclerotic plaques [56].

Aldosterone upregulates gene expression involved in cholesterol efflux via the PPARγ–LXRα–ABCG1 axis and impairs macrophage apoptosis and efferocytosis through MR overexpression, and hence further promoting foam cell accumulation [57]. It also enhances macrophage infiltration into atherosclerotic plaques by upregulating intercellular adhesion molecule-1 (ICAM-1) and monocyte chemoattractant protein-1 (MCP-1) [58,59].

Aldosterone stimulates the production of pro-inflammatory cytokines, including interleukin-6 (IL-6) and tumour necrosis factor-alpha (TNF-α), in both the myocardium and vascular smooth muscle cells [60,61]. These cytokines intensify plaque inflammation and protease activity, facilitating matrix degradation and increasing the risk of plaque rupture [60]. Aldosterone also promotes the release of placental growth factor (PlGF), which activates leukocytes within coronary vessel walls [62].

Together, these inflammatory and atherogenic effects contribute to early atherosclerosis and heightened plaque vulnerability, ultimately increasing the risk of myocardial infarction and stroke as manifestations of end-organ damage in PA [61,63].

### 6.3. Atrial Fibrillation and Other Cardiac Arrhythmias

Since the publication of a meta-analysis highlighting the increased risk of atrial fibrillation (AF) in individuals with PA, subsequent studies have reported a high prevalence of PA among patients presenting to emergency departments with AF [4,64]. In a prospective study of 411 patients with newly diagnosed hypertension and unexplained AF, approximately 20% were found to have PA [64].

Excess aldosterone likely predisposes individuals to cardiac arrhythmias by disrupting electrolyte balance and altering ion channel function in cardiomyocytes [65]. Under normal physiological conditions, aldosterone helps regulate sodium, potassium, and calcium currents to maintain cardiac action potential and contractility [17]. However, in states of aldosterone excess, ion channel expression and function are dysregulated, resulting in electrophysiological instability. Specifically, aldosterone induces calcium overload in cardiomyocytes through upregulation of L-type and T-type voltage-gated calcium channels, leading to prolonged P-wave duration and increased right atrial conduction time, hallmarks of AF [65,66].

Another key mechanism contributing to AF in PA is aldosterone-induced cardiac fibrosis [67,68]. Calcium overload activates profibrotic pathways in atrial tissue, leading to fibrosis and atrial dilation, which promote the development of permanent AF [65]. Aldosterone also stimulates cardiac fibroblast proliferation and collagen deposition, contributing to adverse atrial remodelling [18,19]. Additionally, aldosterone reduces matrix metalloproteinase-13 (MMP-13) activity, impairing collagen degradation [66]. These fibrotic changes result in scar formation, disrupting normal electrical conduction and creating areas of slow or re-entrant conduction, which further increase AF risk [67,68]. Importantly, mineralocorticoid receptor antagonists (e.g., eplerenone) can reverse atrial fibrosis and dilation, reducing the progression to persistent AF [69].

Aldosterone promotes renal potassium excretion, increasing the risk of hypokalaemia in patients with PA [70,71]. Severe hypokalaemia can prolong the QT interval and significantly raise the risk of life-threatening arrhythmias such as Torsades de Pointes [72,73,74].

### 6.4. Left Ventricular Hypertrophy

Left ventricular (LV) hypertrophy may result from aldosterone excess, prolonged untreated hypertension, or a combination of both [4,48,75]. Individuals with PA have a higher likelihood of developing LV hypertrophy compared to those with essential hypertension, even when matched for BP, suggesting an independent adverse effect of aldosterone excess [4,48]. A retrospective study of 1186 individuals with PA from Japan demonstrated that nadir aldosterone levels after confirmatory tests correlate strongly with the severity of LV hypertrophy after adjusting for age and blood pressure [76]. In one study, treatment with low-dose spironolactone over three years led to regression of LV hypertrophy in patients with PA, with more than half (57%) achieving normalisation [77].

Aldosterone promotes cardiomyocyte hypertrophy via activation of intracellular signalling cascades, including the mitogen-activated protein kinase (MAPK) pathway, which increases protein synthesis and cell growth [20]. It also induces hypertrophy through upregulation of cardiotrophin-1 (CT-1), a pro-hypertrophic cytokine [78]. In patients with untreated hypertension, LV hypertrophy may initially arise as a compensatory mechanism in response to increased cardiac workload from elevated systolic pressure [75]. However, studies have demonstrated that the degree of LV hypertrophy in PA correlates with the severity of autonomous aldosterone secretion and is disproportionate to the hemodynamic load, reflecting a maladaptive process [79].

Chronic LV hypertrophy is a known risk factor for heart failure and arrhythmias, underscoring the importance of early detection and targeted treatment in patients with PA [75].

### 6.5. Cardiac Fibrosis and Heart Failure

Aldosterone excess contributes to cardiac stress and injury through increased oxidative stress and upregulation of pro-inflammatory cytokines [51]. In response to myocardial injury, aldosterone stimulates cardiac fibroblasts to secrete collagen and other extracellular matrix proteins to repair damaged tissue [18,19,61]. It also promotes inflammation and fibrosis via activation of the circadian protein CLOCK pathway [80]. These maladaptive responses lead to myocardial fibrosis, which in turn increases the risk of heart failure, including both heart failure with reduced ejection fraction (HFrEF) and heart failure with preserved ejection fraction (HFpEF) [61,81].

HFrEF typically results from myocardial infarction and subsequent cardiac dyskinesis or akinesis, but can also arise from non-ischaemic causes, including dilated cardiomyopathy and chronic pressure overload [82]. In contrast, HFpEF, is a heterogenous syndrome that often associates with comorbidities such as obesity, hypertension and metabolic dysfunction [83]. While HFpEF could be the result of pathological ventricular remodelling and fibrosis due to chronic exposure to aldosterone excess, it can also be the consequence of LV hypertrophy due to chronic pressure overload [84]. Aldosterone-induced fibrosis impairs myocardial relaxation during diastole, contributing to HFpEF and diastolic dysfunction [81,84,85]. Individuals with PA (n = 76) exhibited lower global circumferential peak diastolic strain rate, a marker of diastolic dysfunction, and evidence of diffuse myocardial fibrosis on T1 mapping of cardiac MRI compared to individuals with essential hypertension (n = 27), even after adjusting for age and hypertension duration [86]. In the cardiac MRI study that evaluated extracellular volume (ECV) on T1 mapping, another marker for diffuse myocardial fibrosis, has shown that individuals with PA (n = 20) have higher ECV fraction than those with essential hypertension (29.5% vs. 23.3%) [87]. There is a good concordance between CMR-derived ECV and histological ECV fraction [88], and mice models with hypertension and LV hypertrophy have demonstrated that ECV expansion can be prevented by spironolactone [89]. Moreover, plasma aldosterone concentrations were inversely associated with post-contrast T1 time, indicating a higher burden of interstitial myocardial collagen deposition [86].

Aldosterone exacerbates symptoms in individuals with pre-existing heart failure by promoting sodium retention, fluid overload, and oedema [50]. Emerging research suggests that both HFrEF and HFpEF could be reversed with targeted treatment of PA, highlighting the potential direct pathological role of aldosterone [81,90]. A study of 129 individuals with aldosterone-producing adenomas (lateralizing PA) showed a higher prevalence of LV eccentric hypertrophy and impaired diastolic function, as evidence by higher E/e’ ratio which provides an estimate of the LV filling pressure, compared to BP-matched controls with essential hypertension, and these parameters were reversed following adrenalectomy (E/e’ ratio: pre-surgery 12.5 ± 4.2 vs. post-surgery 11.3 ± 3.7; *p* = 0.002) [81]. A study from Singapore reported that individuals with PA and early sign of LV systolic dysfunction (mean LV ejection fraction 60%), as assessed by global longitudinal strain (GLS), who received 12 months of targeted treatment and reversal of renin suppression had improvement in GLS [91]. Current evidence supports the benefits of targeted PA treatment, even when the initial LV function is preserved [92], especially with unsuppressed renin, but its therapeutic role in overt HFrEF remains unknown and require further evaluation.

## 7. Therapeutic Implications and Future Directions

Overt PA is associated with worse cardiovascular outcomes, but this can be mitigated by timely diagnosis and prompt treatment initiation [4]. Individuals with low renin concentration and inappropriately normal or high aldosterone concentration, who desire surgical treatment, may undergo aldosterone suppression testing followed by lateralisation testing with adrenal vein sampling to identify potentially curable disease [93,94]. Lateralizing PA is mostly caused by aldosterone-producing adenomas (APA) which may be resected to achieve a cure of aldosterone excess. In many patients, remission of PA following adrenalectomy leads to an attenuation or cessation of anti-hypertensive medications to maintain normal BP [95]. Individuals with bilateral PA require lifelong targeted medical therapy using mineralocorticoid receptor antagonists, as well as dietary sodium restriction for optimal BP control and mitigating the CV risk associated with aldosterone excess [93].

The management of aldosterone excess with mineralocorticoid receptor antagonists has been established in clinical practice for over six decades [96]. Since its discovery in 1957, spironolactone remains one of the most commonly prescribed medications for heart failure [96,97,98]. However, due to its steroidal structure, spironolactone is associated with sex hormone-related side effects, including gynaecomastia, menstrual irregularities, and sexual dysfunction, that can impair adherence and lead to premature discontinuation [98,99]. Eplerenone is a widely used alternative to spironolactone due to fewer antiandrogenic effects from its more selective properties, but was found to be less effective in BP control than spironolactone for individuals with PA [100].

Newer non-steroidal mineralocorticoid receptor antagonists have demonstrated comparable efficacy with fewer off-target effects and are gaining traction across cardiovascular and renal indications [92,101,102]. Finerenone, a non-steroidal mineralocorticoid receptor antagonist, has been shown to reduce hospitalisations and cardiovascular mortality in patients with HFpEF or heart failure with mildly reduced ejection fraction (EF 40–50%) [92]. However, evidence in PA remains limited; only one pilot study has compared finerenone to spironolactone in PA, demonstrating similar efficacy [103]. Another promising drug class is aldosterone synthase inhibitors, which selectively block the enzyme required for aldosterone synthesis with negligible effect on cortisol synthesis [104,105,106,107]. One of several agents within this class, Baxdrostat, was recently tested in 15 patients with PA under Phase 2a study compared to placebo [104]. Baxdrostat led to a mean BP reduction of 24.9 mmHg (95% CI 19.0 to 30.8) and 90.9% (95% CI 73.9 to 92.9) reduction in plasma aldosterone concentration over 12 weeks [104]. In addition to PA, these agents have also reduced blood pressure in individuals with resistant hypertension [105,106] and reduced albuminuria in those with CKD [107].

The recognition of subclinical PA as a contributor to arterial stiffness and adverse cardiac remodelling in middle-aged adults, and to early signs of LV damage in young adults, is particularly concerning for normotensive individuals, who are rarely screened for aldosterone excess [9,11,36,42]. Currently, no evidence supports the use of mineralocorticoid receptor antagonists in normotensive adults with subclinical PA to reduce cardiovascular risk. However, given that the harmful effects of mild aldosterone excess may begin in early adulthood and persist over decades, clinical trials are urgently needed to determine whether mineralocorticoid receptor antagonists can improve long-term cardiovascular outcomes in normotensive individuals with elevated ARR [9,11,36,42].

Emerging evidence underscores the association between subclinical PA and cardiovascular damage, suggesting that even mildly elevated aldosterone levels can have cumulative adverse effects [6,7,9,11,36,42]. Although these individuals do not meet formal diagnostic criteria for PA, their chronic exposure to elevated aldosterone may predispose them to long-term cardiovascular harm. Beyond known associations with low birth weight, which has been linked to elevated aldosterone and higher BP in childhood and adolescence, there is limited understanding of early-life determinants of aldosterone dysregulation [28]. Further research is needed to elucidate how early life events influence aldosterone production and PA risk in adulthood.

## 8. Conclusions

Aldosterone excess contributes to adverse cardiovascular outcomes across the lifespan. However, in the absence of hypertension or heart failure, there is currently no evidence to support the routine use of aldosterone blockade for subclinical aldosterone excess. With the emergence of novel therapeutic options, such as non-steroidal mineralocorticoid receptor antagonists and aldosterone synthase inhibitors, clinical trials are needed to determine whether treatment of aldosterone excess in normotensive individuals can improve long-term cardiovascular outcomes. In parallel, greater emphasis on systematic screening for PA in hypertensive populations is essential to facilitate earlier diagnosis and targeted therapy, with the potential to reduce the substantial and often underrecognized cardiovascular burden associated with this condition.

## Data Availability

No new data were created or analysed in this study. Data sharing is not applicable to this article.
